# Utilization of lactic acid in human myotubes and interplay with glucose and fatty acid metabolism

**DOI:** 10.1038/s41598-018-28249-5

**Published:** 2018-06-29

**Authors:** Jenny Lund, Vigdis Aas, Ragna H. Tingstad, Alfons Van Hees, Nataša Nikolić

**Affiliations:** 1 Department of Pharmaceutical Biosciences, School of Pharmacy, University of Oslo, Oslo, Norway; 2 Department of Life Sciences and Health, Faculty of Health Sciences, OsloMet - Oslo Metropolitan University, Oslo, Norway

## Abstract

Once assumed only to be a waste product of anaerobe glycolytic activity, lactate is now recognized as an energy source in skeletal muscles. While lactate metabolism has been extensively studied *in vivo*, underlying cellular processes are poorly described. This study aimed to examine lactate metabolism in cultured human myotubes and to investigate effects of lactate exposure on metabolism of oleic acid and glucose. Lactic acid, fatty acid and glucose metabolism were studied in myotubes using [^14^C(U)]lactic acid, [^14^C]oleic acid and [^14^C(U)]glucose, respectively. Myotubes expressed both the MCT1, MCT2, MCT3 and MCT4 lactate transporters, and lactic acid was found to be a substrate for both glycogen synthesis and lipid storage. Pyruvate and palmitic acid inhibited lactic acid oxidation, whilst glucose and α-cyano-4-hydroxycinnamic acid inhibited lactic acid uptake. Acute addition of lactic acid inhibited glucose and oleic acid oxidation, whereas oleic acid uptake was increased. Pretreatment with lactic acid for 24 h did not affect glucose or oleic acid metabolism. By replacing glucose with lactic acid during the whole culturing period, glucose uptake and oxidation were increased by 2.8-fold and 3-fold, respectively, and oleic acid oxidation was increased 1.4-fold. Thus, lactic acid has an important role in energy metabolism of human myotubes.

## Introduction

Lactate is produced from glucose through glycolysis and the conversion of pyruvate by lactate dehydrogenase (LDH). Once considered a waste product of anaerobic glycolysis, lactate is now recognized to be formed continuously also under fully aerobic conditions^1^. The main function of lactate is to serve as a precursor of hepatic gluconeogenesis, but also as an energy substrate for aerobic oxidation via the citric acid cycle (TCA cycle) in many peripheral tissues. In a recently published systematic analysis of the fluxes of circulating metabolites in mice, lactate was revealed as a primary source of carbon for the TCA cycle, demonstrating that the contribution of glucose to the TCA cycle in majority of tissues (except brain) occurs through circulating lactate^2^. The major organ of lactate production and utilization is skeletal muscle, especially during exercise. While substantial amount of research has been devoted to investigate lactate metabolism under various conditions *in vivo*^3–6^, underlying cellular processes are still not well described, and studies are mainly limited to cell lines^7,8^. Expanding knowledge on the cellular metabolism of lactate, as well as its impacts on fuel utilization of other energy substrates in skeletal muscle cells, is of importance, as these processes will affect whole-body energy homeostasis.

Depending on the level of contractile activity, energy consumption in skeletal muscles may vary considerably, with an estimated 1000-fold difference between rest and maximal contraction^9^. Energy required for cellular functions, including contraction, is provided by ATP hydrolysis to ADP and P_i_. There are three main mechanisms to generate ATP in skeletal muscle; creatine kinase activity, glycolysis and mitochondrial oxidative phosphorylation. The different muscle fiber types differ in their metabolic preferences. Slow muscles (type I fibers) rely mainly on oxidative phosphorylation, whereas fast muscles (type II fibers) are primarily glycolytic. Plasma lactate concentrations increase rapidly during exercise, and the traditional idea has been that lactate is a waste product that is transported to the liver, kidneys or other organs for clearance^1^. However, it is now generally accepted that lactate is also taken up by the muscles and oxidized^10^. In fact, the contribution of muscles to total body lactate clearance is considerable during exercise^10–12^. Most lactate (75–80%) is disposed of immediately within the tissue or released for reuptake by working muscle^13^. Even in resting muscles, when partial pressure of oxygen is more than sufficient for oxidative phosphorylation to occur, concentrations of lactate and pyruvate are 1.0 mM and 0.1 mM, respectively^14^. After vigorous exercise, but still at oxygen pressure sufficient for maximal mitochondrial respiration, lactate to pyruvate concentration ratio may increase to 500 fold^14^. Thus, glycolysis proceeds to lactate production under both aerobic and anaerobic conditions, since equilibrium constant of LDH strongly favors lactate production^14^. Lower oxygen supply will inhibit oxidative phosphorylation, in which case production of lactate will exceed the rate of oxidative metabolism of pyruvate, resulting in higher lactate concentrations. A well-described effect of endurance exercise in skeletal muscles is increased mitochondrial content, which then serves as a larger sink for pyruvate. Increased mitochondrial oxidative activity requires lower levels of stimulators (ADP), and since some glycolytic enzymes are activated by the same stimulators, glycolysis will be reduced^14^. Thus, lactate is always formed in the process of glycolysis, but the amount produced and transported out of the cell is affected by several factors such as glycolysis rate, activity of oxidative enzymes, oxygen availability and activity of lactate transporters.

Lactate transport across the plasma membrane is mediated by proton-linked monocarboxylate transporters (MCTs), that belong to the SLC16 gene family^15^. The driving force for the lactate transport includes both trans-membrane concentration gradient and local proton availability. Of the 14 identified MCTs, MCTs 1–4 are the most characterized, playing important metabolic roles in most tissues, where MCT1 and MCT4 are the most important isoforms in skeletal muscles^15^. Detailed molecular mechanisms involved in the MCT regulation are still unclear, but probably include both transcriptional and post-transciptional regulations. Of importance is the upregulation of muscle MCT1 expression by exercise and MCT4 expression by hypoxia, implying important role of the latter transporter in cells relying on anaerobic glycolysis for energy production^16^. MCT1 has a higher affinity for lactate than MCT4, and is therefore thought to be more central in lactate uptake, whereas MCT4 probably is more suited for lactate extrusion^17^. MCT1 has been found predominantly in oxidative muscles, where it is required for lactate to enter the cells and be oxidized as an energy fuel^15^. Indeed, expression of MCT1 in skeletal muscles is strongly associated with oxidative capacity and mitochondrial content^15^. MCT4 is widely expressed, especially in tissues relying on glycolysis even when oxygen is available (aerobic glycolysis)^15,18^, where it mainly serves to export lactic acid. In skeletal muscle, MCT1 is upregulated by exercise and chronic stimulations, and well-trained subjects present higher MCT1 content than less trained subjects, which tend to express more MCT4^17^. Furthermore, a 9-week exercise intervention on sedentary individuals increased mitochondrial MCT1 content of *musculus vastus lateralis*^19^. Protein contents of MCT4 have been reported to change only occasionally in response to exercise^20–23^. Hypoxia, on the other hand, is a potent stimulator of MCT4 (but not of MCT1 or MCT2), and this effect is mediated by hypoxia-inducible factor 1α (HIF-1α)^16^. Regulation of MCT4 expression under normoxic conditions is not clarified. MCT2 and MCT3 are less studied, but MCT2 is present in skeletal muscle and is primarily involved in import of lactate^24^. MCT3 is the least described of the four transporters, expression of which is confined to the retinal pigment epithelium and choroid plexus^16^. Higher abundance of MCT3 in fast-twitch glycolytic and fast-twitch oxidative than in slow-twitch muscle fibers has been reported^25^. As opposed to MCT2, MCT3 mainly facilitates efflux of lactate^25^.

Energy substrate preference in skeletal muscle is variable, and during the fed state, increased availability of plasma glucose stimulates glucose oxidation and fatty acid synthesis, whereas fatty acid oxidation increases both during fasting and sustained exercise^26,27^. However, when exercise intensity increases, the fuel preference shifts from fatty acid to glucose metabolism^28,29^. The ability to switch between energy substrates is thought to be a characteristic of healthy human myotubes. Adaptability refers to the cells ability to increase fatty acid oxidation with an increase in fatty acid availability. Suppressibility refers to the suppressive effect acutely added glucose has on the oxidation of fatty acids. Accordingly, metabolic inflexibility is defined as loss of ability to readily switch from fatty acid oxidation during fasting to glucose oxidation in the postprandial state^30^, and has also been associated with obesity and type 2 diabetes^30,31^. It has been shown that metabolic switching might be an intrinsic characteristic of human skeletal muscle cells^32^, and we have shown in other studies that metabolic switching of human myotubes could be changed by altering the extracellular milieu^33–36^. Role of lactate in metabolic switching of skeletal muscle cells has, to our knowledge, not been described.

The purpose of the present work was to study lactate metabolism in cultured human myotubes. The ability of human myotubes to use lactate as an energy source, but also as a substrate for storage as glycogen and intracellular lipids, was investigated. Additionally, effects of acute and chronic lactate exposure on the metabolism of the two other main energy substrates in human skeletal muscle cells, oleic acid and glucose, were studied.

## Results

### Lactate as an energy substrate for myotubes

**Figure 1:**
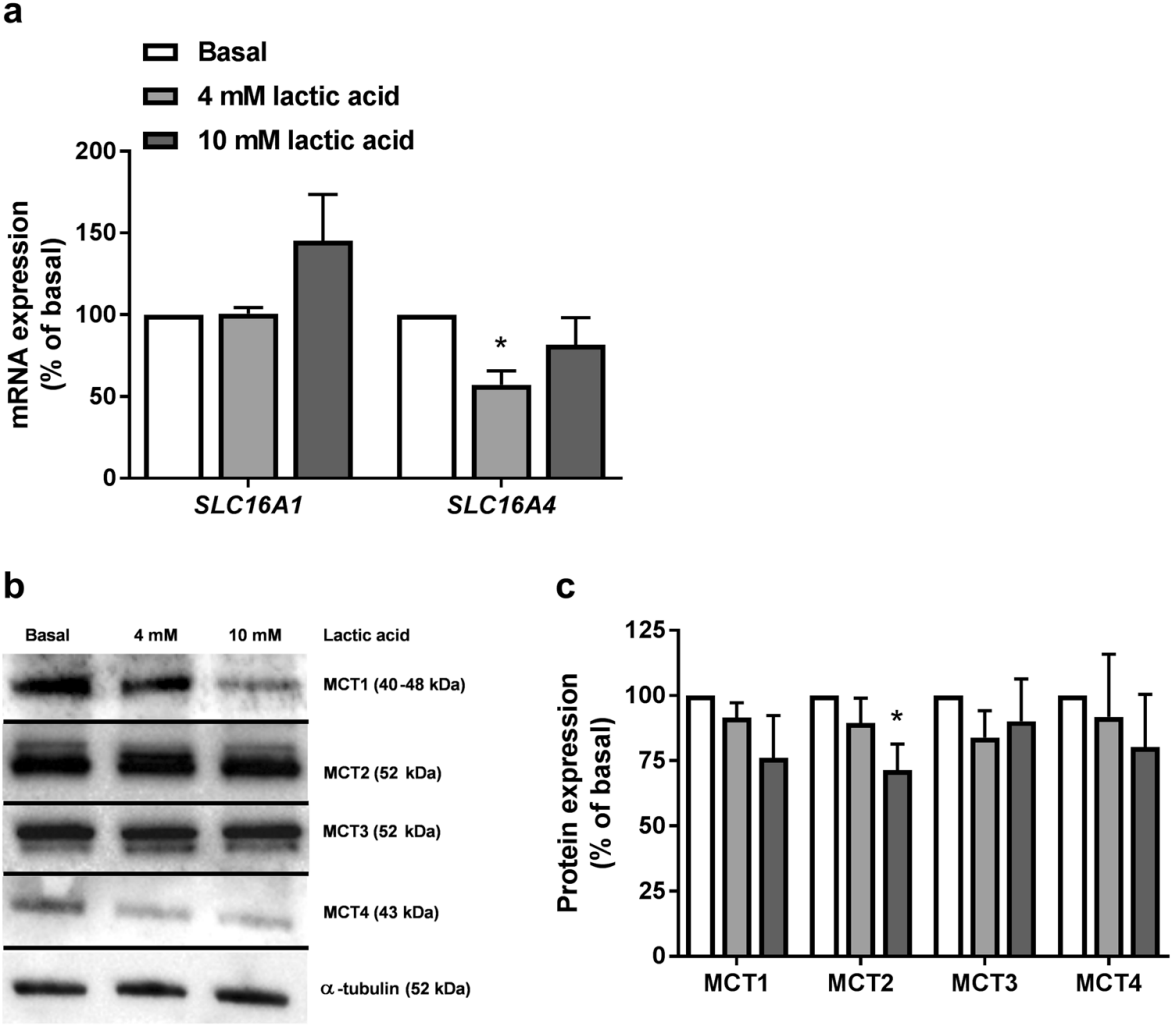
mRNA and protein expression of lactate transporters. Satellite cells were cultured and differentiated to myotubes. They were kept in normal differentiation medium (basal, 5.5 mM glucose) or exposed to lactic acid (4 mM or 10 mM replacing glucose) for 24 h before total cell RNA and protein content were isolated. (**a**) mRNA expressions of *SLC16A1* (MCT1) and *SLC16A4* (MCT4). All values were corrected for the housekeeping gene acidic ribosomal phosphoprotein P0 (*RPLP0*), and presented as means ± SEM relative to basal (n = 5). (**b**,**c**) Protein expressions of MCT1–4. (**b**) One representative immunoblot. (**c**) Quantified immunoblots relative to basal. All values were corrected for the housekeeping control α-tubulin, and data are presented as means ± SEM relative to basal (n = 5). Dividing lines delineate blots from different gels. The samples derive from the same experiment, and all blots were processed in parallel. Full-length blots are presented in Supplementary Fig. 1. *Statistically significant vs. basal (*p* < 0.05, paired Student’s t-test).

Extracellularly added lactate must be transported into muscle cells in order to function as an energy substrate. Expression levels of the two main lactic acid transporters in skeletal muscles, MCT1 (*SLC16A1*) and MCT4 (*SLC16A4*), were studied in fully differentiated myotubes grown in standard differentiation medium (5.5 mM glucose) at both mRNA (Fig. 1a) and protein level (Fig. 1b,c). Two other transporters, MCT2 and MCT3, were studied at protein level (Fig. 1b,c). mRNA expression level of MCT4 (*SLC16A4*) was reduced in cells treated with 4 mM lactic acid (Fig. 1a), while protein levels of other MCTs were unaffected after 24 h of lactic acid pretreatment, except for MCT2, which was reduced in cells treated with 10 mM lactic acid (Fig. 1b,c).

**Figure 2:**
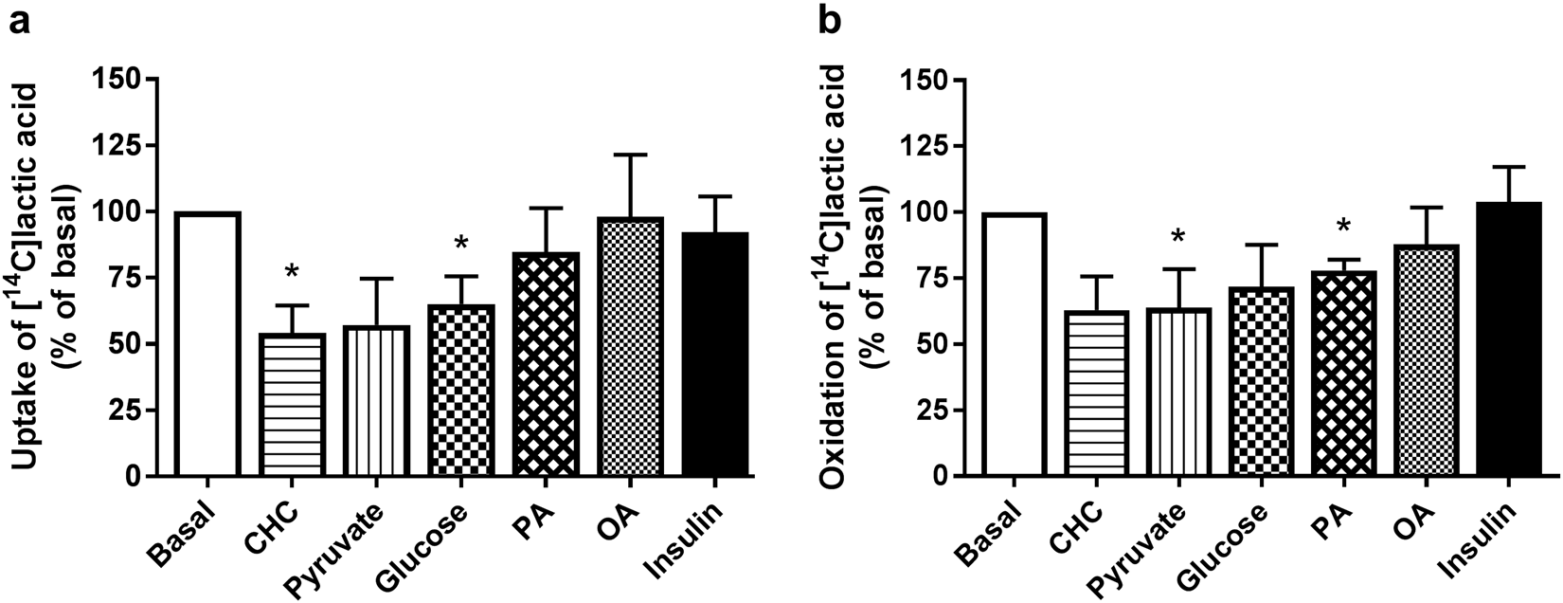
Lactate metabolism in human myotubes and effects of insulin, other energy substrates and an inhibitor of monocarboxylate transporters. Lactic acid metabolism in human myotubes and the influence of acutely added insulin (100 nM), glucose (5 mM), pyruvate (5 mM), oleic acid (OA, 100 µM), palmitic acid (PA, 100 µM) or α-cyano-4-hydroxycinnamic acid (CHC, 1 µg/ml). Myotubes were incubated with [^14^C(U)]lactic acid (1 µCi/ml, 100 µM) for 4 h. (**a**) Uptake of lactic acid was assessed as the sum of oxidized [^14^C]lactic acid (CO_2_) and remaining cell-associated radioactivity (CA) radioactivity. Basal absolute value representing 100% (mean ± SEM): 11.7 ± 5.3 nmol/mg protein. (**b**) Oxidized [^14^C]lactic acid (CO_2_) was trapped in a filter and counted by liquid scintillation. Basal absolute value representing 100% (mean ± SEM): 5.3 ± 2.6 nmol/mg protein. All data are presented as means ± SEM, relative to basal (n = 4–6). *Statistically significant vs. basal (*p* < 0.05, paired Student’s t*-*test).

Further, lactate metabolism was studied by incubating myotubes with [^14^C]lactic acid (1 µCi/ml, 100 µM) for 4 h (Fig. 2). Lactic acid was clearly taken up by the cells (11.7 ± 5.3 nmol/mg protein) (Fig. 2a), and 45 ± 4% (5.3 ± 2.6 nmol/mg protein) of the amount taken up was completely oxidized to CO_2_ (Fig. 2b). Insulin (100 nM) did not stimulate lactic acid uptake or oxidation (Fig. 2a,b, respectively), and cytochalasin B (10 µM) did not inhibit lactic acid uptake (data not shown). However, uptake of lactic acid was reduced in the presence of 5 mM glucose (Fig. 2a), and oxidation was reduced by acute addition of 5 mM pyruvate (Fig. 2b). Palmitic acid (PA, 100 µM) also reduced oxidation of lactic acid (Fig. 2b), whereas oleic acid (OA, 100 µM) had no effect on either uptake or oxidation (Fig. 2a,b, respectively). The monocarboxylate inhibitor α-cyano-4-hydroxycinnamic acid (CHC, 1 µg/ml) inhibited lactic acid uptake to 54 ± 10% of basal uptake (Fig. 2a).

**Figure 3:**
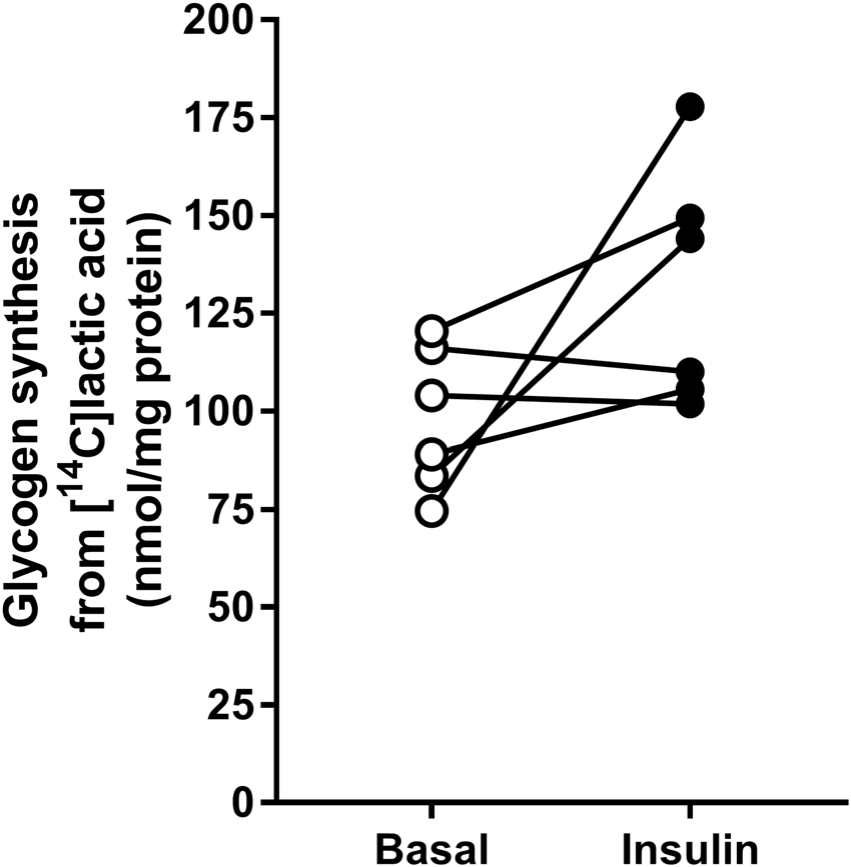
Glycogen synthesis from [^14^C]lactic acid. Myotubes were incubated with [^14^C(U)]lactic acid (1 µCi/ml, 20 mM) the last 24 h of the differentiation period. Glycogen synthesis was measured as incorporation of [^14^C(U)]lactic acid into glycogen in the presence or absence of 100 nM insulin for 3 h. All values are presented as means ± SEM (n = 6). *Statistically significant vs. basal (*p* < 0.05, paired Student’s t*-*test).

In addition to be taken up and oxidized, lactic acid was found to be a substrate for glycogen synthesis in human myotubes (Fig. 3), and insulin increased incorporation of lactic acid into glycogen in four out of six cell donors (Fig. 3).

**Figure 4:**
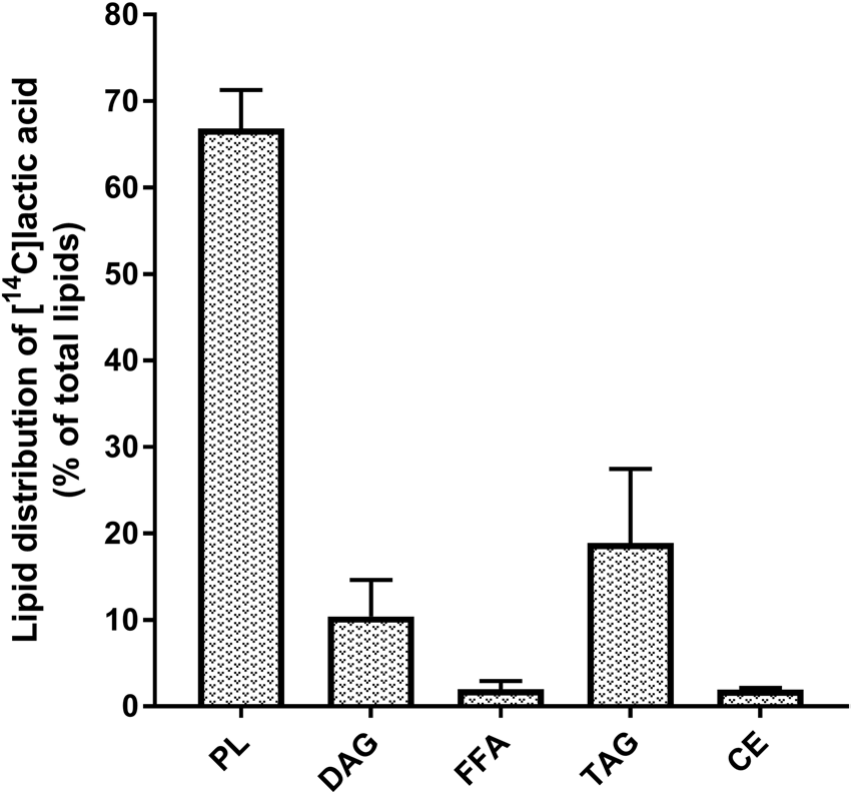
Incorporation of lactic acid into cellular lipids. Myotubes were incubated with [^14^C(U)]lactic acid (1 µCi/ml, 20 mM) for the last 24 h of the differentiation period before total cell lipids were extracted and separated by thin layer chromatography. Incorporation into cellular lipids calculated as % of total lipid content (57.9 ± 29.5 nmol/mg protein). Data are presented as means ± SEM (n = 3). PL, phospholipids; DAG, diacylglycerol; FFA, free fatty acids; TAG, triacylglycerol; CE, cholesteryl ester. *Statistically significant vs. basal (*p* < 0.05, paired Student’s t*-*test).

Lactic acid was also incorporated into cellular lipids (Fig. 4), with a total lipid content of 57.9 ± 29.5 nmol/mg protein. Incorporation into phospholipids (PL) constituted approximately 70% of total lipid content (Fig. 4). Diacylglycerol (DAG) and triacylglycerol (TAG) constituted approximately 10% and 20% of the total lipid content, respectively (Fig. 4), whereas both free fatty acids (FFA) and cholesteryl ester (CE) constituted approximately 2% of the total lipid content (Fig. 4). After hydrolysis of TAG and DAG, 90% of the incorporated lactic acid was recovered in the aqueous (glycerol) phase, whereas 10% was recovered in the organic (fatty acids) phase (data not shown).

### Effects of lactate on the metabolism of glucose and oleic acid

#### Acute effects of lactate exposure

**Figure 5:**
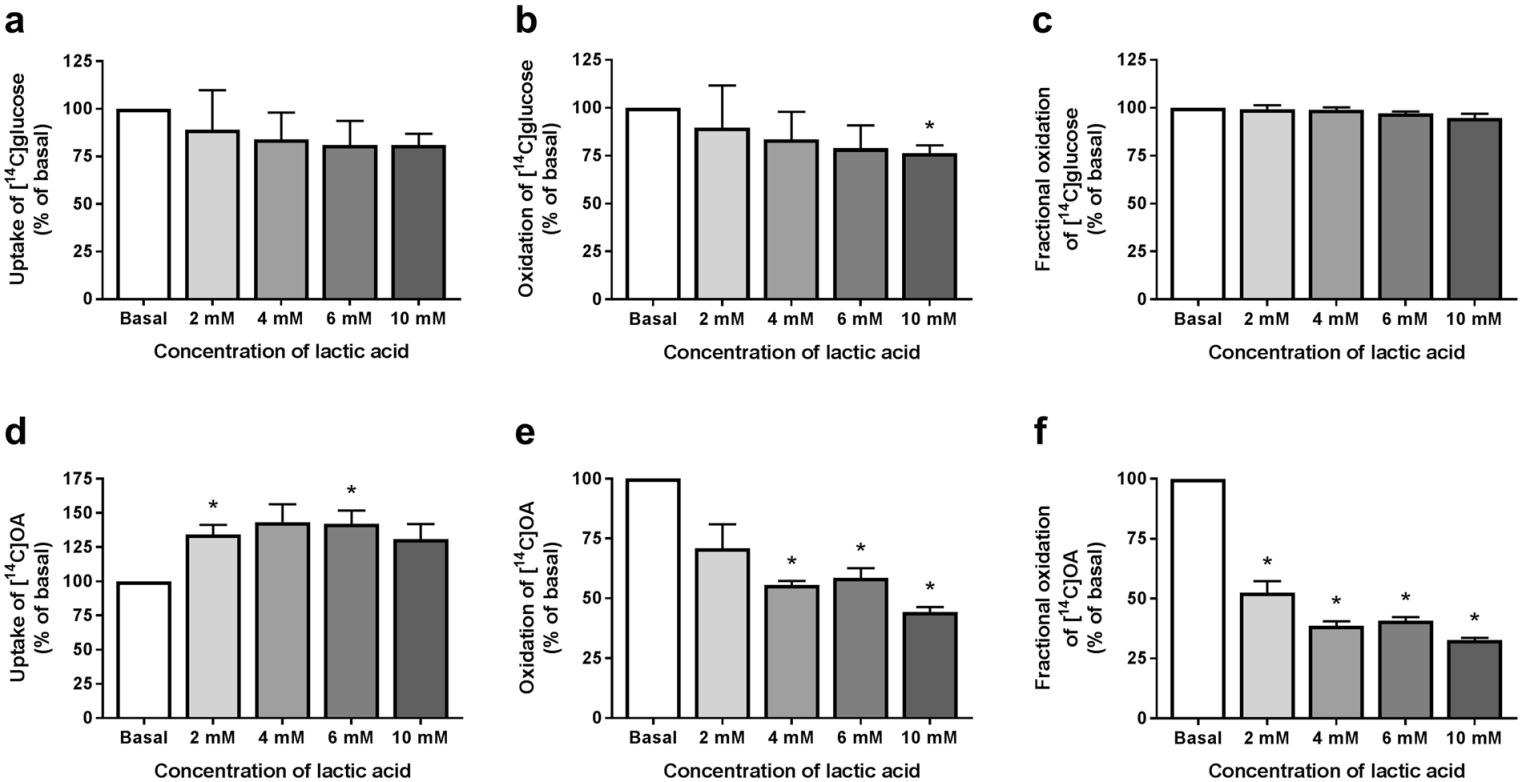
Glucose and oleic acid metabolism after acute addition of lactic acid at different concentrations. Glucose and oleic acid metabolism were studied using [^14^C(U)]glucose (1 µCi/ml, 200 µM) and [^14^C]oleic acid (OA, 1 µCi/ml, 100 µM) for 4 h, respectively, in presence or absence (basal) of lactic acid (2, 4, 6, or 10 mM). Uptake was assessed as the sum of oxidized lactic acid (trapped CO_2_) and remaining cell-associated (CA) radioactivity. Oxidation was measured as CO_2_ trapped in a filter and counted by liquid scintillation. Fractional oxidation was calculated as CO_2_/(CO_2_ + CA). (**a**) Uptake of glucose. Basal absolute value representing 100% (mean ± SEM): 120.9 ± 44.3 nmol/mg protein. (**b**) Oxidation of glucose. Basal absolute value representing 100% (mean ± SEM): 95.9 ± 39.5 nmol/mg protein. (**c**) Fractional oxidation of glucose. (**d**) Uptake of oleic acid (OA). Basal absolute value representing 100% (mean ± SEM): 164.7 ± 47.1 nmol/mg protein. (**e**) Oxidation of OA. Basal absolute value representing 100% (mean ± SEM): 38.3 ± 14.0 nmol/mg protein. All data are presented as means ± SEM relative to basal (n = 3). *Statistically significant vs. basal (*p* < 0.05, paired Student’s t-test).

Acute addition of lactic acid (4 h) did not affect glucose uptake (Fig. 5a), whereas glucose oxidation was decreased when lactic acid was added at the highest concentration (10 mM, Fig. 5b). However, acutely added lactic acid had no effect on fractional glucose oxidation (Fig. 5c). Lactic acid increased oleic acid uptake (Fig. 5d), whereas oleic acid oxidation was markedly reduced (Fig. 5e), causing reduced fractional oleic acid oxidation at all concentrations (Fig. 5f).

#### Chronic effects of lactate exposure

**Figure 6:**
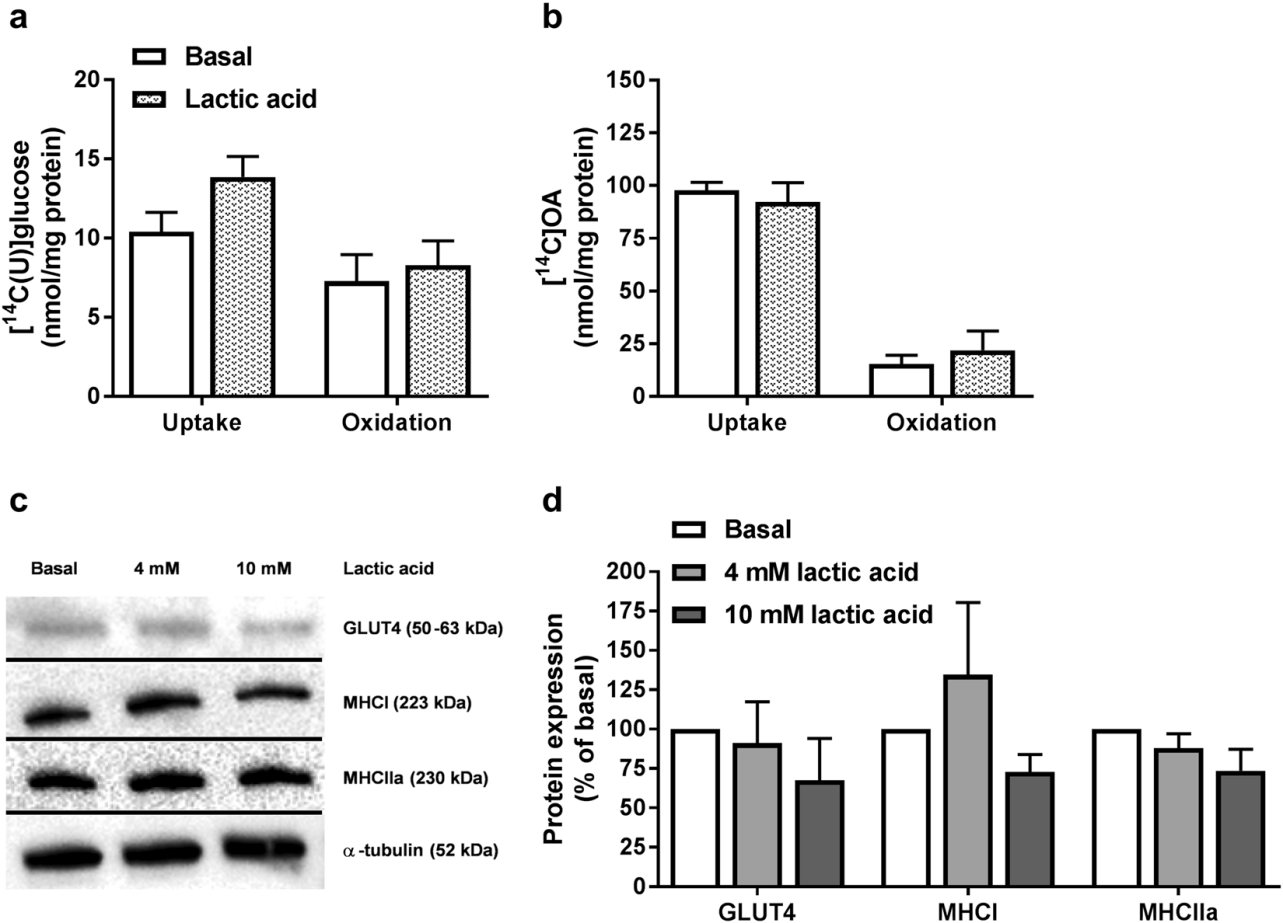
Glucose and oleic acid metabolism, protein expressions of GLUT4, MHCI and MHCIIa after incubation with lactic acid for 24 h. Myotubes were exposed to 5 mM lactic acid or standard glucose containing media (basal, 5.5 mM) for the last 24 h of the differentiation period. (**a**) Uptake and oxidation of [^14^C(U)]glucose (1 µCi/ml, 200 µM) over 4 h. (**b**) Uptake and oxidation of [^14^C]oleic acid (OA, 1 µCi/ml, 100 µM) over 4 h. Oxidation was measured as CO_2_ trapped in a filter and counted by liquid scintillation. Uptake was assessed as the sum of oxidized (trapped CO_2_) glucose or oleic acid and remaining cell-associated (CA) radioactivity. Data are presented as means ± SEM relative to basal (n = 3). (**c**,**d**) Protein expressions of GLUT4, MHCI and MHCIIa assessed by immunoblotting. (**c**) One representative immunoblot. (**d**) Quantified immunoblots relative to basal. All values were corrected for the housekeeping control α-tubulin, and data are presented as means ± SEM relative to basal (n = 5). Dividing lines delineate blots from different gels. The samples derive from the same experiment, and all blots were processed in parallell. Full-length blots are presented in Supplementary Fig. 6.

Chronic exposure to lactic acid (5 mM for 24 h), compared to chronic exposure to the same concentration of glucose, revealed a tendency towards enhanced glucose uptake (Fig. 6a), whereas oleic acid metabolism was unaffected (Fig. 6b). Furthermore, protein expressions of the glucose transporter GLUT4, as well as the fiber type markers MHCI (type I fibers) and MHCIIa (type II fibers) were unaffected by lactic acid (4 mM or 10 mM) exposure for 24 h (Fig. 6c,d).

#### Effects of prolonged lactate exposure

**Figure 7:**
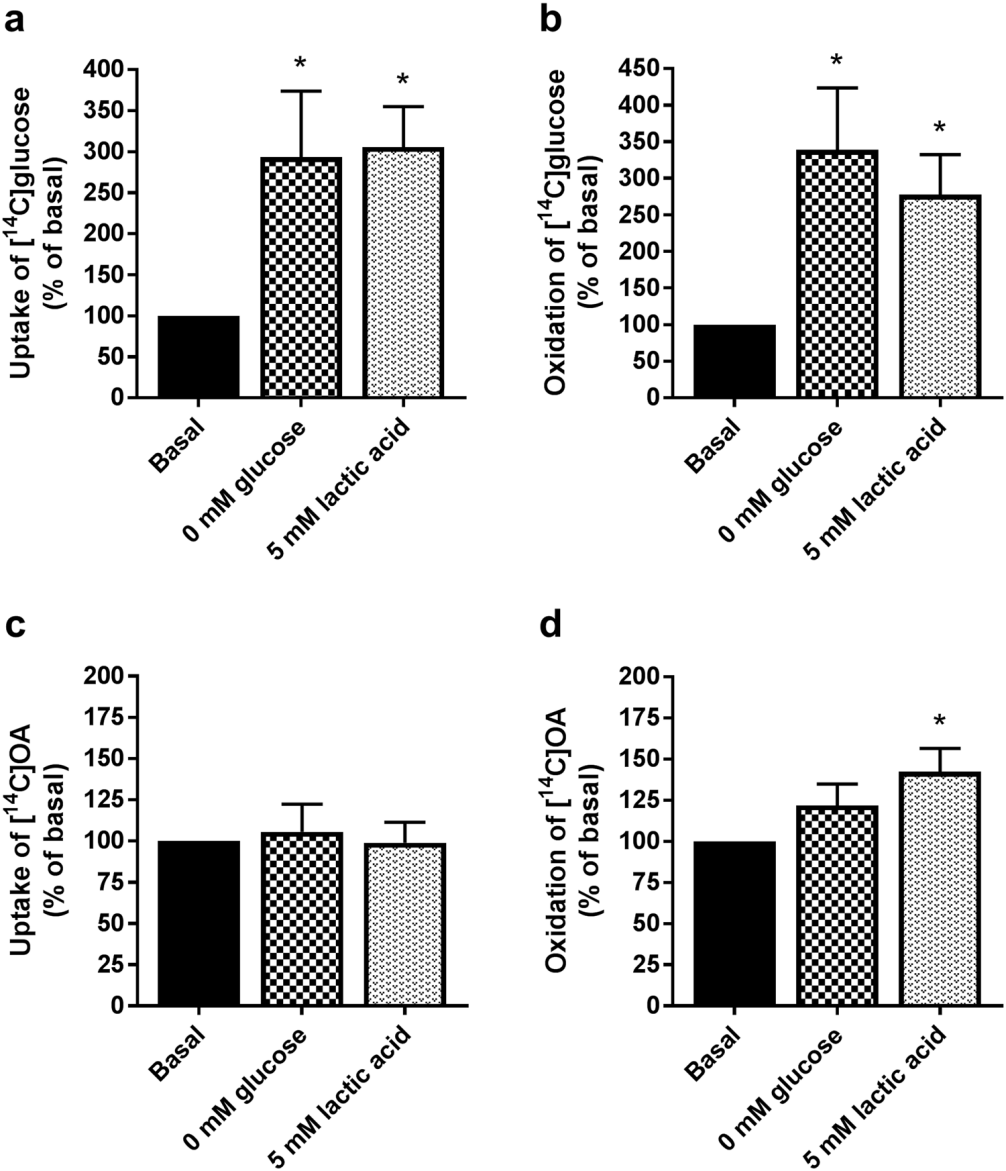
Glucose and oleic acid metabolism after culturing myotubes in cell media where glucose was replaced by lactic acid or removed. Myotubes were cultured in regular cell media (basal, 5.5 mM glucose), cell media containing no glucose (0 mM glucose) or cell media where glucose were replaced with lactic acid (5 mM) for the whole proliferation and differentiation period. Oxidation was measured as CO_2_ trapped in a filter and counted by liquid scintillation. Uptake was assessed as the sum of oxidized (trapped CO_2_) lactic acid and remaining cell-associated (CA) radioactivity. (**a**,**b**) Uptake and oxidation of [^14^C(U)]glucose (1 µCi/ml, 200 µM). (**c**,**d**) Uptake and oxidation of [^14^C]oleic acid (OA, 1 µCi/ml, 100 µM). Absolute values (means ± SEM) representing 100%: glucose uptake; 16.7 ± 2.6 nmol/mg protein, glucose oxidation; 8.3 ± 0.8 nmol/mg protein, oleic acid (OA) uptake; 77.8 ± 12.9 nmol/mg protein and oleic acid (OA) oxidation; 7.1 ± 1.8 nmol/mg protein. Data are presented as means ± SEM relative to basal (n = 11). *Statistically significant vs. basal (*p* *<* 0.05, paired Student’s t-test).

When increasing the exposure time by replacing glucose with lactic acid during the whole proliferation and differentiation period; both glucose uptake (Fig. 7a) and oxidation (Fig. 7b) were increased. Oleic acid uptake (Fig. 7c) was not affected, but oxidation was enhanced (Fig. 7d). The effect of lactic acid was comparable to removing glucose completely (0 mM glucose, Fig. 7). Replacing glucose with lactic acid only during the differentiation period (7 days) did not affect oleic acid metabolism (data not shown).

**Figure 8:**
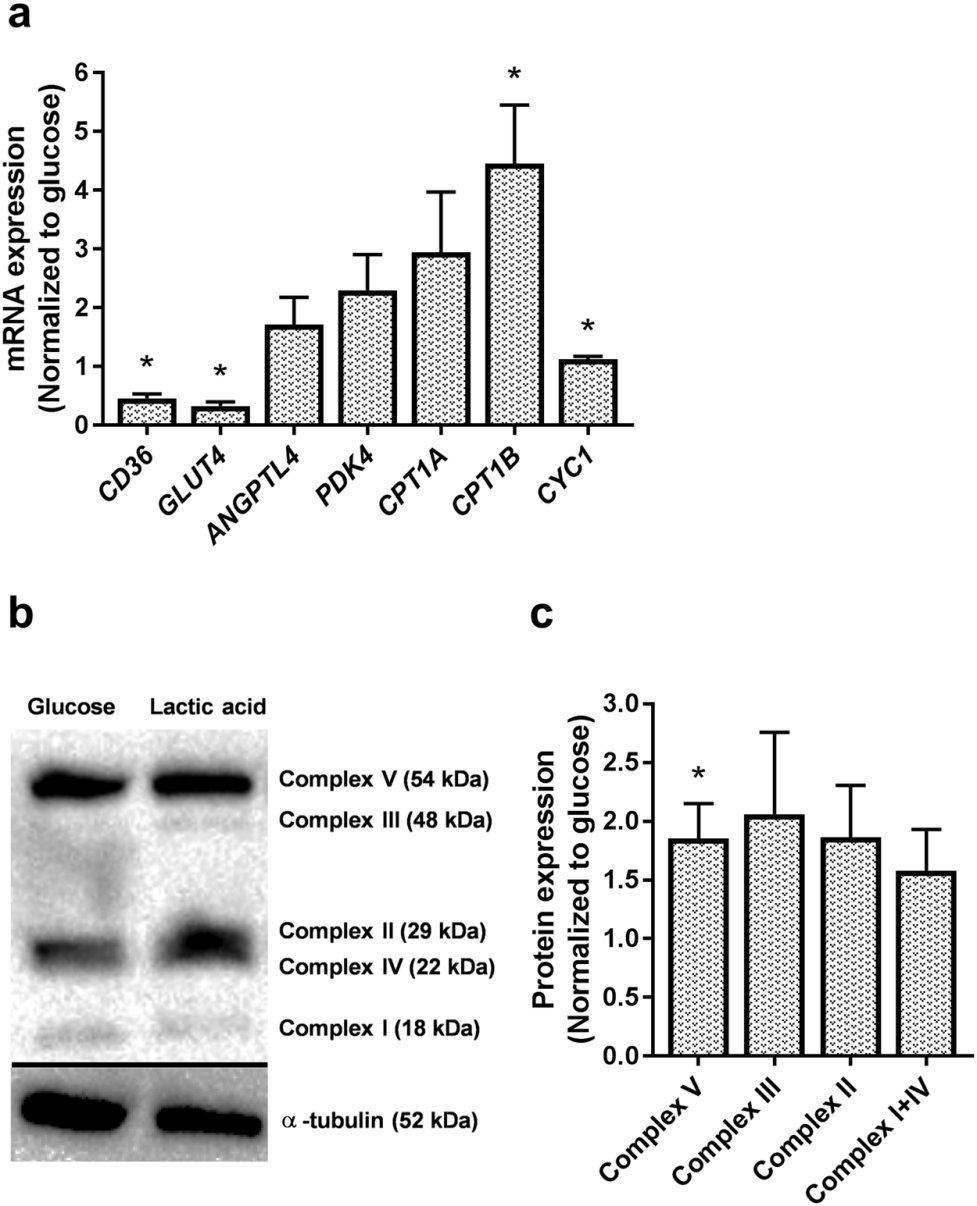
mRNA and protein expressions of factors involved in regulation of metabolism in myotubes cultured in a lactic acid-containing medium. Myotubes were cultured in standard cell media (basal, 5.5 mM glucose) or cell media where glucose had been replaced with lactic acid (5 mM) for the whole proliferation and differentiation period. After ended differentiation, total cell RNA and protein contents were isolated. (**a**) mRNA expressions of CD36 molecule (*CD36*), solute carrier family 2 member 4 (*GLUT4*), angiopoietin like 4 (*ANGPTL4*), pyruvate dehydrogenase kinase 4 (*PDK4*), carnitine palmitoyl transferase 1 (*CPT1A and B)* and cytochrome c1 (*CYC1*). All values were corrected for the housekeeping control *RPLP0*, and presented as means ± SEM relative to basal (n = 5). (**b**,**c**) Expressions of proteins involved in mitochondrial phosphorylation, OXPHOS (complex I subunit NDUFB8, complex II subunit, complex III subunit core 2, complex IV subunit II and complex V (ATP synthase subunit α)). (**b**) One representative immunoblot. (**c**) Quantified immunoblots normalized to glucose. All values were corrected for the housekeeping control α-tubulin, normalized to glucose, and data are presented as means ± SEM relative to basal (n = 5). Dividing lines delineate blots from different gels. The samples derive from the same experiment, and all blots were processed in parallell. Full-length blots are presented in Supplementary Fig. 8. *Statistically significant vs. basal (*p < *0.05, paired Student’s t*-*test).

To further investigate underlying mechanisms in conditions where glucose was replaced by lactic acid for the entire culturing period, we studied mRNA and protein expressions of several factors involved in metabolic processes in skeletal muscles (Fig. 8). Compared to the cells grown in glucose-containing media, mRNA expressions of the transporters of glucose and fatty acids, solute carrier family 2 member 4, *GLUT4* and *CD36*, respectively, were decreased in cells cultured in lactate-containing media (Fig. 8a). On the other hand, carnitine palmitoyl transferase 1B (*CPT1B*) and cytochrome c1 (*CYC1*), involved in the transport of fatty acids across the mitochondrial membrane and mitochondrial function, respectively, were increased in lactate-cultured cells (Fig. 8a). mRNA expression levels of *ANGPTL4*, a potent inhibitor of lipoprotein lipase activity induced in the fasting state by peroxisome proliferator activated receptors (PPARs)^37^, *CPT1A* and pyruvate dehydrogenase kinase 4 (*PDK4*), an inhibitor of the pyruvate dehydrogenase complex, were not changed in cells cultured in the presence of lactate (Fig. 8a). Protein expression of the electron transport chain’s complex V (ATP synthase subunit α) was also increased in the presence of lactic acid (Fig. 8b).

Despite the observed changes in the metabolism of both glucose and fatty acids, the metabolic flexibility of myotubes grown and differentiated in lactic acid-containing media did not differ from cells grown in standard glucose-containing medium, assessed as glucose suppression of oleic acid oxidation (suppressibility) and the ability to increase oleic acid oxidation with increasing oleic acid concentration (adaptability) (data not shown).

## Discussion

The aim of the present study was to explore the ability of cultured human myotubes to utilize lactate as a fuel source, but also to investigate effects of lactate on the metabolism of the two other main energy substrates, glucose and oleic acid. Lactate is no longer considered as only a waste product of glycolysis due to hypoxia, but also an important factor in cellular and whole body metabolism. Glucose and fatty acids are the two most important fuel sources of skeletal muscle. Moreover, skeletal muscle is the largest producer of lactate. Thus, shedding light on the impact of lactate on cellular metabolism of glucose and fatty acids in skeletal muscle cells is of particular interest for understanding whole-body energy balance, and has, to our knowledge, not been thoroughly explored. Our main finding was that isolated human skeletal muscle cells have the ability to metabolize lactate in terms of uptake, oxidation and storage not only in form of glycogen, but also as intracellular lipids. Furthermore, prolonged exposure to lactic acid increased glucose metabolism and oleic acid oxidation. These findings were accompanied by increased mRNA expression levels of *CPT1B* and *CYC1*, as well as higher protein expression of complex V (ATP synthase subunit α) of the mitochondrial respiratory chain, suggesting an overall improvement in mitochondrial function of myotubes exposed to lactic acid for an extended period.

The ability of the cells to take up extracellularly added lactate was first verified by showing expression of the monocarboxylate transporters responsible for lactate transport in skeletal muscles, MCT1 and MCT4, both at mRNA and protein level, and MCT2 and MCT3 at protein level. It has recently been shown in C2C12 cells that 16 mM lactate induced an increase in mRNA expression levels of both *SLC16A1* (MCT1) and *SLC16A4* (MCT4), but with different times of peak induction, suggesting different dynamics in the expression of the two transporters upon lactate stimulation^38^. We observed lower mRNA expression of *SLC16A4* in cells pretreated with 4 mM lactic acid for 24 h, but not with 10 mM lactic acid. Lactate concentrations may reach 10–20 mM in the circulation^39,40^, or even 40 mM in the skeletal muscle after intense anaerobic exercise in humans, which has been shown to increase expression of both lactate transporters^41^. In another *in vivo* study, a progressive decrease in mRNA expression levels of both MCT1 and MCT4 over the period of 9–72 h after a single bout of exercise was reported^42^. Interestingly, in the same study, protein content of MCT1 was higher after 24–72 h compared to 9 h post-exercise, while MCT4 remained unchanged^42^, suggesting post-transcriptional modifications. Proteins and mRNA expressions may have distinct kinetics, which emphasizes importance of time points when taking biopsies post-exercise or cell-harvesting to interpret adaptive responses. Our finding of decreased mRNA level of MCT4 at 4 mM of lactic acid is supportive to *in vivo* results, and in the present study, both protein and RNA isolation was performed immediately after ended lactic acid treatment. The fact that the observed decrease in mRNA expression of *SLC16A4* at 4 mM of lactic acid was not seen when concentration was increased to 10 mM is somewhat unexpected. However, it has been shown that MCT4 is upregulated *in vitro* by hypoxia-inducible factor-1α pathway, and hypoxia during exercise has been suggested to induce transient mRNA bursts of MCT4, followed by subsequent reduction in mRNA contents^43^. Thus, discrepancies between mRNA and protein levels, as well as inconsistencies regarding effects of exercise *in vivo* studies have been reported earlier, suggesting complex dynamics and post-transcriptional modifications that are yet to be described. The concentrations used in this study were in the range 2–10 mM, and at these conditions, no differences in mRNA expression of *SLC16A1* or protein expressions of MCT1 or MCT4 were observed. As mentioned in the introduction, MCT1 has predominantly been found in oxidative fibers, whereas MCT4 is more abundant in glycolytic fibers^19,44^. Cultured human myotubes are generally limited with regards to their reflection of the fiber type of the muscle they are isolated from, and tend to mainly express fiber type markers of fast, glycolytic fibers^45^. It has also been shown that peroxisome proliferator activated receptor gamma coactivator −1α (PGC-1α) is a strong inducer of both MCT1 (but not MCT2 and MCT4) expression and uptake of lactic acid in muscles^46^. Expression of PGC-1α, which is a contraction-induced regulator of oxidative mitochondrial function, is reduced in cultured human myotubes^47^. Low expression of PGC-1α could partially explain lack of increase in protein expression of MCT1 in our study. We further found that both MCT2 and MCT3 were expressed in cultured human myotubes. Interestingly, protein level of MCT2 was reduced in myotubes exposed to the highest concentration of lactic acid (10 mM). Whether MCT2 adapts to chronically increased muscle activity is still not defined, although it was reported in one study that it is not regulated by PGC-1α, nor does it change in response to contractions^46^. MCT2 is expressed in skeletal muscles and it is primarily involved in import of lactic acid, with a higher affinity for both lactic acid and pyruvate than MCT1^46^. This makes it a suitable transporter in tissues taking up large amounts of lactic acid as a fuel. In spite of that, expression of MCT2 does not appear to correlate to oxidative capacity of different muscles, nor is it more abundant in oxidative muscles, in contrast to MCT1^46^. Mechanisms of regulation of MCT2 are also not clarified. MCT3, which is even less described, was also expressed in myotubes in the present study, but protein expression of this transport was unaffected by different concentrations of lactic acid. Higher abundance of MCT3 in fast-twitch glycolytic and fast-twitch oxidative than in slow-twitch muscle fibers has been reported, and it appears to be involved in the efflux of lactic acid^25^. It is important to keep in mind that *in vivo*, most muscles contain a mixture of both primarily glycolytic and primarily oxidative muscle fibers, and the proportion of each fiber type will depend on many factors from genetics to whether that particular muscle uses high-intensity exercise (glycolytic) or endurance exercise (oxidative). Thus, both expression and regulation of the MCTs is probably tightly associated with the particular muscle and its metabolic activity.

The fact that all four transporters were expressed in the cells suggests that cultured myotubes should be able to transport lactate across membranes. This was confirmed by flux studies of labelled lactate, which was shown to be taken up by the cells, and approximately 45% was further oxidized to CO_2._ This is in accordance with the *in vivo* findings, where it has been shown that muscle fibers can take up lactate for subsequent metabolic use of intramuscular lactate^48,49^. Oxidative muscle fibers predominantly oxidize lactate, whereas glycolytic fibers primarily convert lactate to glycogen^50–53^. A probable pathway of intracellular lactate disposal involves conversion to pyruvate followed by entry into the Krebs cycle^54^. We further show that, in addition to being oxidized, lactate can be incorporated and stored as glycogen as well. This is in accordance with *in vivo* studies; glyconeogenesis from lactate has been observed in human muscles^55^. Studies on rat muscle showed both glyceroneogenesis and glyconeogenesis from lactate, and reverse flux of pyruvate to phosphoenolpyruvate was found to be the common route^56^. Indeed, during exercise, lactate is by far the most important gluconeogenic precursor in humans, as it is during fasting^57^.

Further, we showed that lactate can be incorporated in complex intracellular lipids. Chen *et al*. showed that lactate was metabolized to lipids in cultured HeLa cells and H460 human lung cancer cells^58^, and Jin *et al*. showed in studies on rat muscle that lactic acid could be converted to glycerol^56^. It has also been reported that lactate, at physiological concentrations, can induce triglyceride accumulation in adipocytes of both humans, mice and rats^59^, and recently, a dose-dependent lactate-induced induction of triglyceride content was demonstrated in C2C12 cells at concentrations of 16 mM and 20 mM^38^. As far as we know, incorporation of lactate to lipids has never before been observed in human skeletal muscle cells. While the majority of lactic acid was incorporated into phospholipids in the present study, partition to TAG was also substantial. Of the lactic acid incorporated in DAG and TAG, 90% was recovered in the aqueous (glycerol) phase, whereas 10% was recovered in the organic (fatty acid) phase.

We further found that pyruvate decreased both uptake and oxidation of lactic acid, as would be expected of a pyruvate metabolite. Glucose was also found to decrease both uptake and oxidation of lactate, which is in line with reports of human myotubes being highly glycolytic when grown in presence of glucose^60^, in addition to having low mitochondrial oxidative capacity resembling fast (glycolytic) muscle fibers^61^. Cultured human myotubes’ reliance on glucose as favorable fuel is also demonstrated by our observations that higher concentrations of acutely added lactate were necessary to suppress glucose oxidation than oleic acid oxidation (10 mM vs. 4 mM, respectively). Palmitic acid also caused a decrease in lactate oxidation. The monocarboxylic acid transport inhibitor CHC caused, as expected, a decrease in lactate uptake. Insulin had no effect on lactate metabolism and cytochalasin B did not inhibit lactate uptake, implying an uptake mechanism independent of translocation of transporters.

Acutely added lactate (4 h) suppressed both glucose and oleic acid metabolism, which is in agreement with a previous study where increased plasma lactate was associated with a decline in plasma FFAs, an anti-lipolytic effect on adipose tissue, and an inhibitory effect on muscle fat oxidation^54^. Chronic treatment (24 h) with 5 mM lactic acid, on the other hand, had no effect on either glucose or oleic acid metabolism; neither did it affect expression of the glucose transporter *GLUT4*. In these cells, glucose in the culturing media was replaced with the same concentration of lactate (5 mM) for a period of 24 h prior to harvesting of the cells, but not during the experiment. Therefore, possible explanations for the lack of effects may be too short pretreatment and absence of lactate during the 4 h course of experiment.

To increase exposure time to lactate, cells were exposed to either lactate or glucose for the entire culturing period. At these conditions, both glucose uptake and oxidation were increased, whereas oleic acid oxidation was increased without a corresponding effect on uptake. Thus, by replacing glucose with lactate for the entire culturing period, we induced an increase in the oxidative metabolism of the cells, which was mainly reflected as an increase in glucose metabolism. Based on these results, one may suggest that lactate forces the cells towards a state of energy deprivation and thereby increases the oxidative capacity of the cells, as metabolism levels were fairly similar as after culturing in media containing no glucose.

To shed light on the underlying mechanisms of the observed effects, we studied protein and mRNA expression levels of several metabolically important factors. Compared to the cells grown in glucose-containing media, mRNA expressions of the transporters of glucose and fatty acids, *GLUT4* and *CD36*, respectively, were decreased in cells cultured in presence of lactic acid. On the other hand, expressions of two important markers of mitochondrial function and fatty acid oxidation, *CYC1* and *CPT1B*, respectively, were both increased after prolonged exposure to lactate, as was protein expression of complex V (ATP synthase subunit α) of the mitochondrial respiratory chain. These data generally support functional results and suggest an increased transport of the fatty acids taken up across the inner mitochondrial membrane, an improved mitochondrial function and establishment of a more oxidative cell model in general. However, decreased level of *GLUT4* was in contrast to the observed increase in glucose uptake, but this observation can probably be explained by generally low *GLUT4* expression in skeletal muscle cells *in* vitro^62^, and the fact that basal glucose uptake can mainly be mediated by other glucose transporters (GLUT1 and 3) in primary human myotubes^63,64^. Discrepancies between *GLUT4* levels and glucose uptake have also been reported previously^47^.

We have previously observed a remodeling of oxidative energy metabolism by replacing glucose with galactose as carbohydrate source during growth and differentiation^65^. In general, myotubes became more oxidative and seemed to utilize glucose better than oleic acid, which we suggested implied an improved metabolic switching^65^. In the present study, however, none of the parameters of metabolic switching were affected in cells that had been cultured under conditions where glucose was replaced by lactate. Thus, although replacing glucose with lactate had a positive effect on both oleic acid and glucose oxidation, the metabolic flexibility of the cells was unaffected.

In conclusion, cultured human skeletal muscle cells are able to take up, oxidize and store lactate in complex lipids and glycogen. Further, we have found that lactate exposure affects the metabolism of glucose and oleic acid in cultured human myotubes, and this impact appears to be related to the duration of exposure to lactate. These findings suggest an important role of lactate in energy metabolism of human skeletal muscle cells.

## Materials and methods

### Materials

Dulbecco’s Modified Eagle’s Medium (DMEM-Glutamax™) low glucose, DMEM without glucose, fetal bovine serum (FBS), sodium pyruvate, Dulbecco’s Phosphate Buffered Saline (DPBS) without Mg^2+^ and Ca^2+^, penicillin/streptomycin (10000 IE/ml), amphotericin B, and trypsin-EDTA were from Gibco Invitrogen (Gibco, Life Technologies, Paisley, UK). Ultroser G was from Pall (Cergy-Saint-Christophe, France) and insulin (Actrapid^®^ Penfill^®^ 100 IE/ml) was from Novo Nordisk (Bagsvaerd, Denmark). SkBM-kit (SkGM) and BioWhittaker^®^ Phosphate Buffered Saline (PBS) were from Lonza (Wakersville, MD, US). VWR^®^ Grade 703 Blotting Paper was from VWR (Poole, UK). Bovine serum albumin (BSA, essentially fatty acid free), L-carnitine, D-glucose, oleic acid (OA, 18:1, n-9), palmitic acid (PA, 16:0), sodium lactic acid, trypan blue 0.4% solution, HEPES, DMSO, L-glutamine, gentamicin, cytochalasin B, α-cyano-4-hydroxycinnamate (CHC), protease inhibitor, phosphatase II inhibitor, β-mercaptoethanol, and Ponceau S solution were from Sigma-Aldrich (St.Louis, MO, US). D-[^14^C(U)]glucose (2.9 mCi/mmol), [1-^14^C]oleic acid (53 mCi/mmol and 56.3 mCi/mmol) and L-[^14^C(U)]lactic acid (118.4 mCi/mmol and 150.6 mCi/mmol) were provided by PerkinElmer^®^ (Boston, MA, US). [^14^C(U)]glucose (10 mCi/mmol) was from American Radiolabeled Chemicals, Inc. (St. Louis, MO, US). 96-well Corning^®^ CellBIND^®^ tissue culture plates were from (Schiphol-Rijk, the Netherlands). Unifilter^®^−96 GF/B, 96-well Isoplate^®^, OptiPhase Supermix, and TopSeal^®^-A transparent film were from PerkinElmer (Shelton, CT, US). Nunc™ Cell Culture Treated Flasks with Filter Caps, Nunc™ 96-MicroWell™ plates, TaqMan reverse transcription kit reagents, High-Capacity cDNA Reverse Transcription Kit, primers for TaqMan PCR, MicroAmp^®^ Optical 96-well Reaction Plate, MicroAmp^®^ Optical Adhesive Film, and Power SYBR^®^ Green PCR Master Mix were obtained from ThermoFisher Scientific (Roskilde, Denmark). Glycerol, Tris-HCl and thin layer chromatography plates were from Merck (Darmstadt, Germany). Amersham™ Protran™ Premium 0.45 µm NC Nitrocellulose Blotting Membrane was from Amersham™ (GE Healthcare, Esbjerg, Denmark). Bio Rad Protein Assay Dye Reagent Concentrate, Clarity™ Western ECL Substrate, Tris/glycine buffer, Tris/glycine/SDS buffer, SDS, Tween 20, brom-phenyl blue, Goat Anti-Rabbit IgG (H + L)-HRP Conjugate (170–6515) secondary antibody, Goat Anti-Mouse IgG (H + L)-HRP Conjugate (170–6516) secondary antibody, and Mini-Protean^®^ TGX™ gels (4–20%) were from Bio-Rad (Copenhagen, Denmark). The antibodies against GLUT4 (sc-53566)^66^, MCT1 (H-1) (sc-365501)^67^ and MCT4 (G-7)^68^ (sc-376465) were from Santa Cruz Biotechnology (Santa Cruz, CA, US). The antibody against MHCI (MAB1628)^69^ was from Millipore (Temecula, CA, US). The human total OXPHOS antibody (ab110411)^65^ and antibodies against MCT2 (ab129290)^70^ and SLC16A8 (ab60333)^70^ were from Abcam (Cambridge, UK). The antibodies against MHCIIa (3403)^71^ and α-tubulin (2144)^72^ were from Cell Signaling Technology Inc. (Beverly, MA, US). QIAshredder and RNeasy Plus Mini Kit were from QIAGEN (Venlo, the Netherlands).

### Ethics statement

The biopsies were obtained with informed written consent and approval by the Regional Committee for Medical and Health Research Ethics South East, Oslo, Norway (reference number: S-04133). The study adhered to the Declaration of Helsinki.

### Human skeletal muscle cell cultures

Satellite cells were isolated as previously described^73^ from the *musculus obliquus internus abdominis* of lean, healthy volunteers. Donors were 40.3 (±3.7) years old, body mass index 23.6 (±0.8) kg/m^2^, fasting glucose 5.1 (±0.2) mM, insulin, plasma lipids and blood pressure within normal range and no family history of diabetes. The cells were cultured in DMEM-Glutamax (5.5 mM glucose) with 2% FCS, 2% Ultroser G, HEPES, penicillin/streptomycin, gentamicin, and amphotericin B until 70–80% confluence. Myoblast differentiation to myotubes was then induced by changing medium to DMEM-Glutamax (5.5 mM glucose) with 2% FCS, 25 pM insulin, penicillin/streptomycin, gentamicin, and amphotericin B. Experiments were performed after 7-8 days of differentiation. During the culturing process the muscle cells were incubated in a humidified 5% CO_2_ atmosphere at 37 °C, and medium was changed every 2-3 days. Growth and differentiation of cells in media containing lactic acid was done by adding DMEM-Glutamax with no glucose, 5 mM L-glutamine, 5 mM lactic acid, 2% FCS, 2% Ultroser G, penicillin/streptomycin, gentamycin, and amphotericin B the day after seeding. At about 70–80% confluence, medium was changed to DMEM with no glucose, 5 mM glutamine, 5 mM lactic acid, 2% FCS, 25 pM insulin, penicillin/streptomycin, gentamycin, and amphotericin B. For comparison, cells were also grown in regular culturing and differentiation media (DMEM-Glutamax, 5.5 mM glucose) without lactic acid.

### Lactate treatments

Different concentrations of lactate were used for the purpose of specific experiments in the present study. Initially, the ability of the myotubes to take up and oxidize lactate was assessed by using L-[^14^C(U)]lactic acid (1 µCi/ml, 100 μM). To verify protein and mRNA expression of the two lactate transporters, MCT1 and MCT4 (representative genes *SLC16A1* and *SLC16A4*, respectively), myotubes were treated with 4 mM or 10 mM lactate for 24 h. Ability to store lactate as glycogen and intracellular lipids, was determined using L-[^14^C(U)]lactic acid (1 µCi/ml, 20 mM) for 24 h. Concentration of 20 mM was decided based on previous studies investigating incorporation of lactate into triglycerides and glycogen^8,51^.

To investigate impacts of lactate exposure on metabolism of glucose and oleic acid, we differed between acute, chronic and prolonged lactate treatment. In the acute treatment (4 h), myotubes were exposed to increasing concentrations of lactate (2 mM, 4 mM, 6 mM and 20 mM), covering the range of concentrations from physiological levels to those seen after exercise *in vivo*^74,75^. In chronic lactate treatment, glucose in the culturing medium was replaced by the same amount of lactate (5 mM) for 24 h. Finally, prolonged exposure was determined as a condition where glucose in the culturing media was completely replaced by the same concentration of lactate (5 mM) for the entire proliferation and differentiation period.

### Substrate oxidation assay

Skeletal muscle cells (7000 cells/well) were cultured on 96-well CellBIND^®^ microplates. Substrate, D-[^14^C(U)]glucose (1 µCi/ml), [1-^14^C]oleic acid (1 µCi/ml) or L-[^14^C(U)]lactic acid (1 µCi/ml), was given during 4 h CO_2_ trapping as described previously^76^. Substrates were added in DPBS with 10 mM HEPES, 10 µM BSA and 1 mM L-carnitine (L-carnitine was only added with oleic acid). Oleic acid was bound to BSA at a ratio of 2.5:1 (this was adjusted for in the substrate medium). A 96-well UniFilter^®^ microplate, soaked with NaOH (1 M), was mounted on top of the CellBIND^®^ plate and produced CO_2_ was trapped during 4 h incubation at 37 °C. CO_2_ production was measured in the presence or absence of various compounds: insulin (100 nM), glucose (5 mM), oleic acid (100 µM), palmitic acid (100 µM), pyruvate (5 mM), cytochalasin B (10 µM), or CHC (1 µg/ml). CO_2_ production and cell-associated (CA) radioactivity were assessed using a PerkinElmer 2450 MicroBeta^2^ scintillation counter. Protein content in each well was determined with the Bio-Rad protein assay using a VICTOR™ *X*4 Multilabel Plate Reader as described previously^77^. The sum of ^14^CO_2_ and CA was considered as total substrate uptake. Fractional complete oxidation was calculated as CO_2_/(CO_2_ + CA).

### Metabolic flexibility parameters

Suppressibility is the ability of the cells to decrease oleic acid oxidation by acutely added glucose, and is calculated as [1 − (oxidation of 100 µM OA at 5 mM glucose/oxidation of 100 µM OA at no glucose added) × 100%]^33^. Adaptability is the ability to increase oleic acid oxidation with increasing oleic acid concentration, and is calculated as [oxidation of 100 µM OA/oxidation of 5 µM OA]^33^.

### Lipid distribution

Myotubes were incubated with L-[^14^C(U)]lactic acid (1 µCi/ml, 20 mM) for 24 h. Myotubes were then washed twice with PBS and harvested with 200 μl of 0.1% SDS. Cellular lipids were extracted, as previously described^78^, from homogenized cell fractions, separated by thin layer chromatography (TLC) and quantified by liquid scintillation (Tri-Carb 1900, PerkinElmer). A non-polar solvent mixture of hexane:diethyl ether:acetic acid (65:35:1) was used to separate the lipids. The amount of lipids was related to total cell protein concentrations. Saponification of the total lipids was performed as described in^79^ with minor modifications. The cellular total lipid extract from DAG and TAG, respectively, were dried and dissolved in 0.75 ml of 30% KOH and heated at 70 °C for 10 min. An equal volume of 95% ethanol was then added and the mixture was heated at 70 °C for 2 h. After cooling, the aqueous fraction was acidified with 3 M HCl drop-wise and extracted three times with 2 ml light petroleum. The organic fraction was evaporated, redissolved in 1 ml of light petroleum, transferred to scintillation vials and quantified as the acyl fraction using the Tri-Carb scintillation counter. The remaining aqueous fraction contained the glyceryl fraction; 0.5 ml aliquot of this sample was taken and measured for radioactivity using the TriCarb scintillation counter.

### Glycogen synthesis

Myotubes were exposed to differentiation media supplemented with L-[^14^C(U)]lactic acid (1 µCi/ml, 20 mM) for 24 h prior to assay start. At assay start, myotubes were exposed to serum-free DMEM supplemented with L-[^14^C(U)]lactic acid (1 µCi/ml, 20 mM), in presence or absence of 100 nM insulin (Actrapid^®^ Penfill 100 IE/ml), for 3 h to measure glycogen synthesis. The cells were washed twice with PBS and harvested in 1 M KOH. Protein content was determined by use of the Pierce BCA Protein Assay Kit, before 20 mg/ml glycogen and more KOH (final concentration 4 M) were added to the samples. Then L-[^14^C(U)]lactic acid incorporated into glycogen was measured as previously described^33^.

### Immunoblotting

Myotubes were cultured in 6-well plates, and two parallell wells of each donor were treated with either 4 mM or 10 mM lactic acid. Samples for immunoblotting were harvested in Laemmli buffer, and aliquots of 15 μg cell protein were electrophoretically separated by SDS-PAGE (Bio-Rad 4–20% Mini Protean^®^ TGX™ precast gels with Tris/glycine buffer) and transferred to nitrocellulose membranes. The membranes were incubated with antibodies against GLUT4 (1:200), MCT1 (1:200), MCT4 (1:200), MHCI (1:10000), MHCIIa (1:1000), and OXPHOS complexes (1:500) overnight. Immunoreactive bands were visualized with Bio-Rad ImmunStar™ WesternC™-kit, detected with Bio-Rad Chemidoc™ XRS + system, and quantified with Image Lab (version 4.0) software. All samples were derived at the same time and processed in parallel. Expression levels were normalized to one sample used as loading control and further normalized to the endogen control α-tubulin (1:1000).

### RNA isolation and analysis of gene expression by qPCR

Human skeletal muscle cells were washed, trypsinized and pelleted before total RNA was isolated using QIAGEN RNeasy Plus Mini Kit according to the supplier’s protocol. Total RNA was reversely transcribed (25 °C for 10 min, 37 °C for 80 min, 85 °C for 5 min) with a High-Capacity cDNA Reverse Transcription Kit and TaqMan Reverse Transcription Reagents using a 2720 Thermal Cycler. Primers were designed using Primer Express^®^ and real-time qPCR was performed using a StepOnePlus Real-Time PCR system. Each target were quantified in triplicates and carried out in a 25 µl reaction volume in accordance with the supplier’s protocol. All assays were run for 44 cycles (95 °C for 15 s followed by 60 °C for 60 s). The transcription levels were normalized to the housekeeping gene acidic ribosomal phosphoprotein P0 (*RPLP0*, acc.no. M17885). The housekeeping gene glyceraldehyde-3-phosphate dehydrogenase (*GAPDH*, acc.no. NM_002046) was also analyzed; there were no differences between normalizing for *RPLP0* or *GAPDH*. The following forward and reverse primers were used at concentration of 30 µM: *ANGPTL4* (acc.no. NM_139314), *CD36* (acc.no. L06850), *CPT1A* (acc.no. L39211), *CPT1B* (acc.no. D1852C12) and *CYC1* (acc.no. NM_001916), *GAPDH*, *GLUT4* (acc.no. M20747), *PDK4* (acc.no. BC040239), *RPLP0, SLC16A1* (acc.no. NM_003051, representing MCT1), and *SLC16A4* (acc.no. NM_004696, representing MCT4).

### Statistics

Data are presented as means ± SEM in nmol/mg protein or as percent of control. The value *n* represents the number of different donors, each with at least duplicate observations. Statistical comparison between different treatments was performed by Student’s t-test using GraphPad Prism 6 for Windows. The parameter of interest was entered as the dependent variable and pretreatment (lactic acid or no glucose) and acute treatments were entered as fixed variables. Differences were considered statistically significant at *p*-values < 0.05.

## Electronic supplementary material

Supplementary information
