# Supplementary material for: Utilization of lactic acid in human myotubes and interplay with glucose and fatty acid metabolism
Source: Sci Rep. 2018 Jun 29;8:9814. doi: 10.1038/s41598-018-28249-5 (PMC6026123; doi:10.1038/s41598-018-28249-5)

Utilization of lactic acid in human myotubes and interplay with glucose and fatty acid metabolism

Jenny Lund[a], Vigdis Aas[b], Ragna H. Tingstad[b], Alfons van Hees[b], Nataša Nikolić[a*]

[a]*Department of Pharmaceutical Biosciences, School of Pharmacy,* University *of Oslo, Oslo, Norway*

[b]*Department of Life Sciences and Health, Faculty of Health Sciences, Oslo and Akershus University College of Applied Sciences, Oslo, Norway*

Supplementary Figure 1

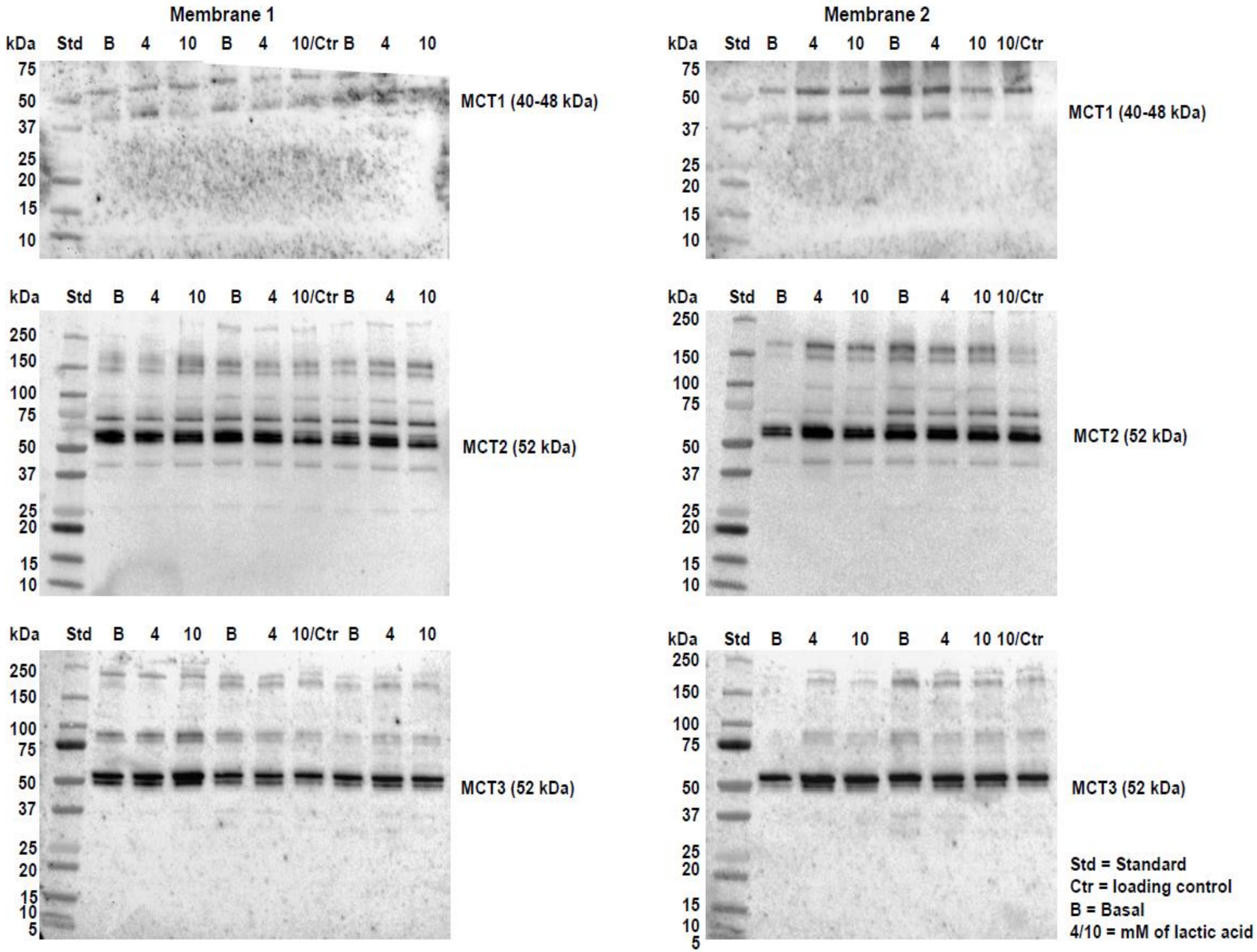

Supplementary Figure 1 (continued)

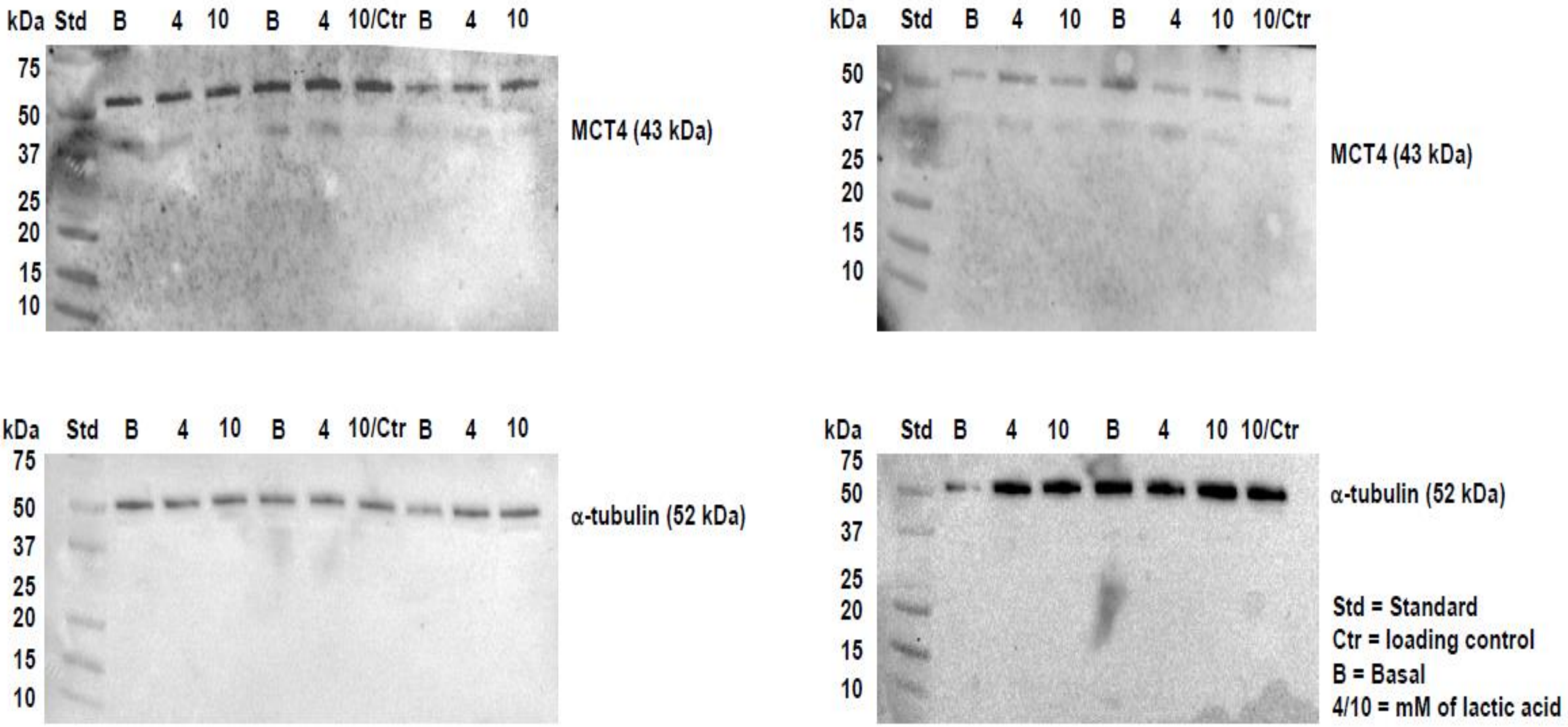

Supplementary Figure 6

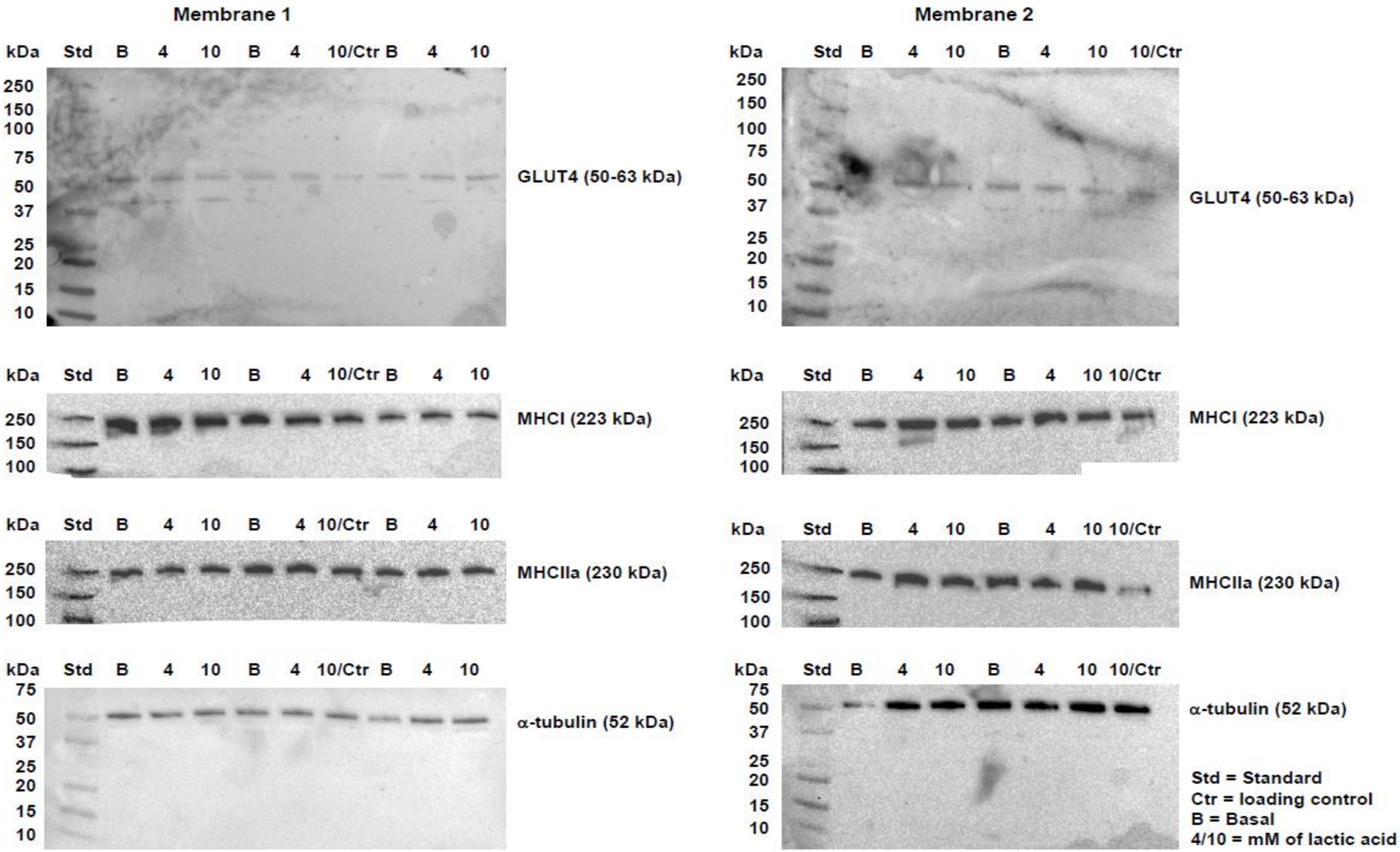

Supplementary Figure 8

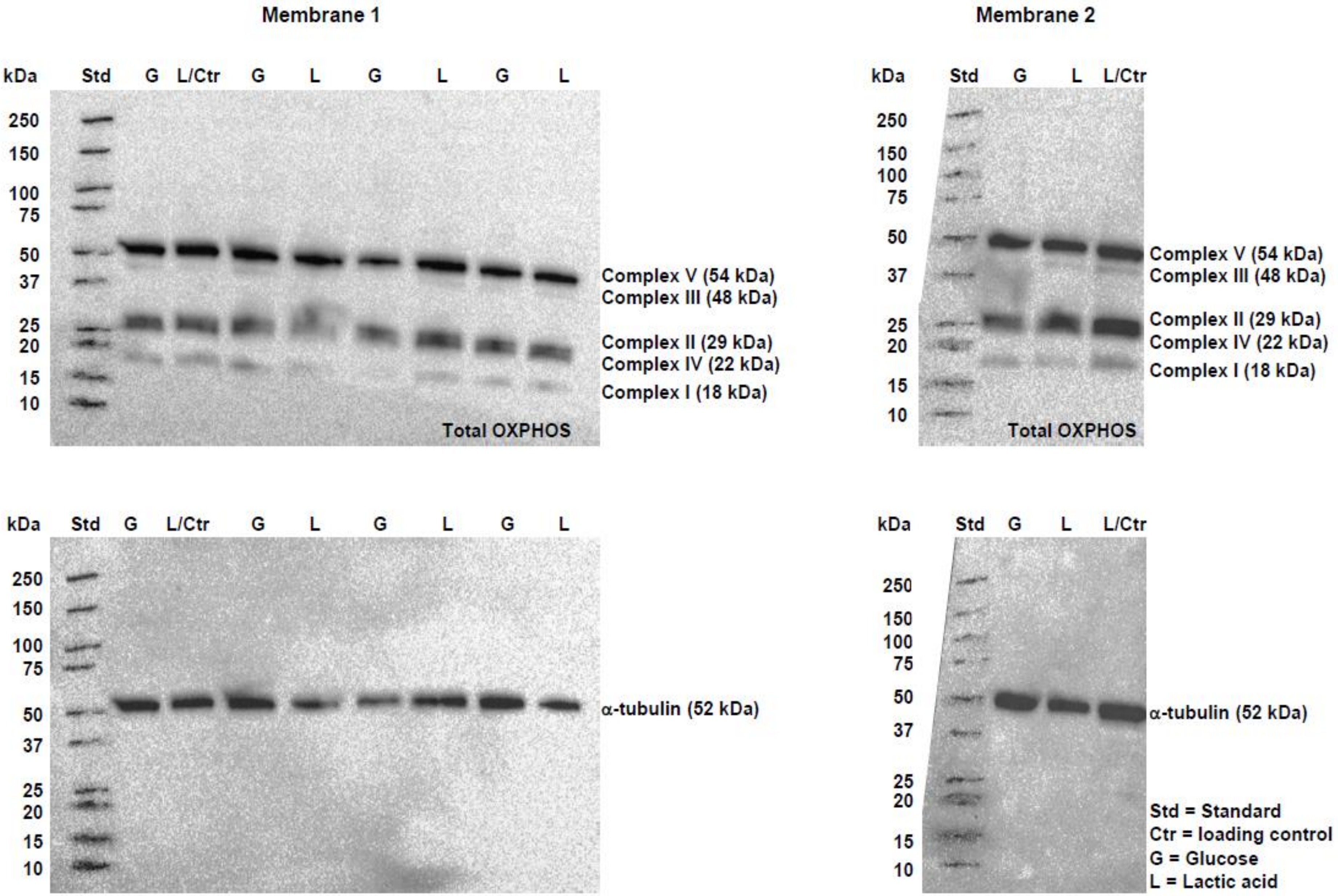

Supplement: Supplementary file 1 — Supplementary information [file 41598_2018_28249_MOESM1_ESM.pdf]
